# Gastric Ischemia and Cecal Necrosis: A Simultaneous Presentation of Two Rare Entities

**DOI:** 10.7759/cureus.4696

**Published:** 2019-05-17

**Authors:** Michael T Quinn, Venu Lakshminarasimhan, Christopher Orpiano, Gurjeet Kang, Joseph Staffetti

**Affiliations:** 1 Gastroenterology, Regional Medical Center Bayonet Point, Hudson, USA

**Keywords:** gastric ischemia, cecal ischemia, right lower quadrant pain, splanchnic, gastrointestinal ischemia, colonic ischemia, superior mesenteric artery stenosis, celiac artery stenosis

## Abstract

A 65-year-old woman presented with right lower quadrant (RLQ) abdominal pain of three days duration. During her hospitalization, she underwent computed tomography (CT) of the abdomen, duplex ultrasound of the abdomen, esophagogastroduodenoscopy (EGD), and colonoscopy as part of a diagnostic workup. The workup identified high-grade obstructions of the celiac artery (CA), superior mesenteric artery (SMA), atypical appearing gastric ulcers, and a diffusely ulcerated cecum, which created a mass-like appearance. The patient developed cecal perforation despite mesenteric vessel stenting and ultimately required right hemicolectomy for definitive management. This case report represents a rare presentation of simultaneous gastric ischemia and cecal ischemia with necrosis in a patient with underlying peripheral vascular disease.

## Introduction

Isolated gastric ischemia and isolated cecal ischemia are uncommon due to an abundance of collateral circulation in the stomach and the colon. Furthermore, such cases are not commonly reported in the literature [1-2]. Isolated right-sided colonic ischemia (IRCI) is a well-known entity, however, it occurs much less frequently than left-sided colonic ischemia (LSCI) does [2]. Isolated cecal necrosis due to ischemia is even less common, and it is an unusual cause of a surgical abdomen [2-4]. Isolated gastric ischemia can be caused by vasculitis, prolonged hypotension, gastric volvulus, and thromboembolism [1-2]. An awareness of these rare conditions will enable clinicians to arrive at a diagnosis and initiate appropriate therapies in a timely manner. The following case details a rare presentation of a patient with simultaneous gastric ischemia and cecal ischemia with necrosis.

## Case presentation

A 65-year-old female presented to the emergency department with complaints of abdominal pain, nausea, and vomiting of three days duration. In the week prior to admission, she had a poor appetite and a 4 lb weight loss. Previous medical history was significant for gastroesophageal reflux disease (GERD), hypertension, peripheral vascular disease, and chronic constipation. Social history was notable for cigarette smoking for several decades. The patient's abdominal pain was predominantly in the right lower quadrant (RLQ). One year prior, she had undergone esophagogastroduodenoscopy (EGD) for further evaluation of abdominal pain; it revealed significant inflammation in the stomach and was labeled as “hemorrhagic gastritis.” Gastric biopsies showed no evidence of infection or malignancy. At that time, colonoscopy to evaluate her abdominal pain and constipation revealed small areas of ulceration in the cecum that on biopsy revealed a fibrinous exudate without necrosis. Random colon biopsies from normal-appearing mucosa were unremarkable. There was no histologic evidence or history of inflammatory bowel disease (IBD). At the time of this most recent hospitalization, her vital signs were within normal limits. Physical exam disclosed a slender and frail-appearing woman in no distress. Abdominal examination revealed a mildly tense abdomen with exquisite tenderness to even gentle palpation in the RLQ. There were no peritoneal signs. Routine laboratory tests revealed leukocytosis with white blood cell (WBC) 19.4x10^3/uL and normal hemoglobin and platelet counts. The basic metabolic panel was normal except for a potassium level of 2.5 mEq/L. The liver chemistry panel, lipase, and troponins were within normal limits. Lactic acid was minimally raised at 2.1 mmol/L and urinalysis revealed no indicators of infection.

**Figure 1:**
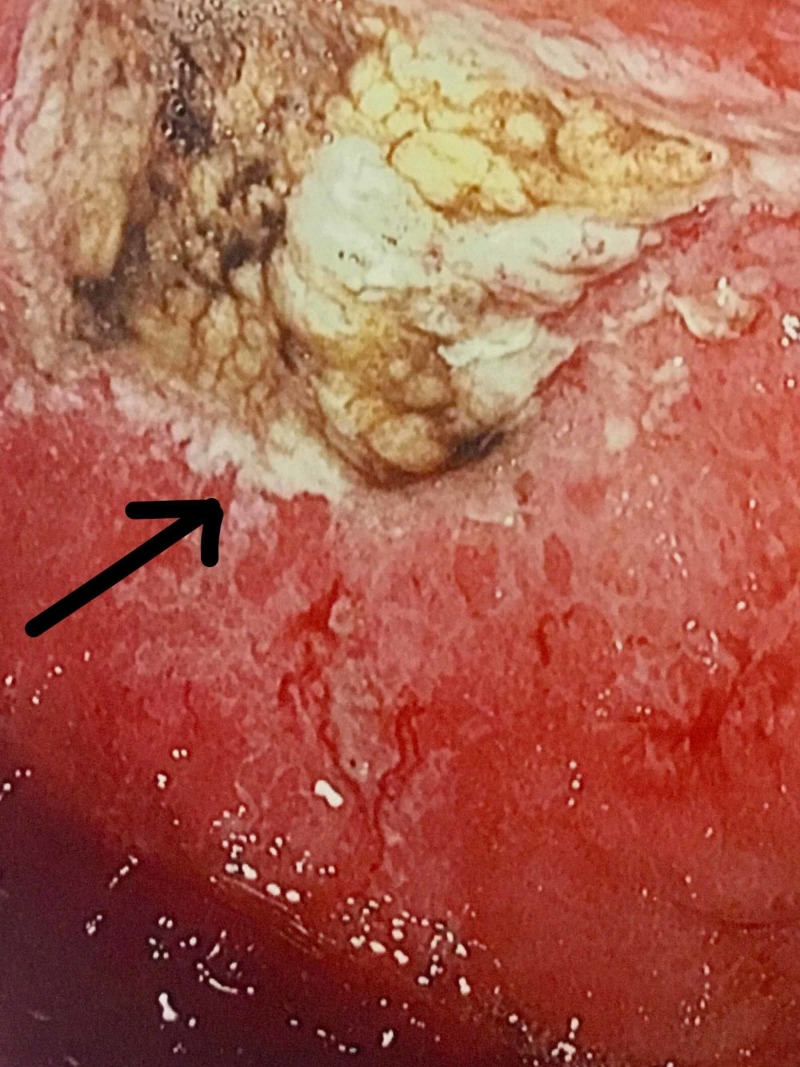
Ischemic gastric ulcer in the antrum as seen on EGD EGD: esophagogastroduodenoscopy

**Figure 2:**
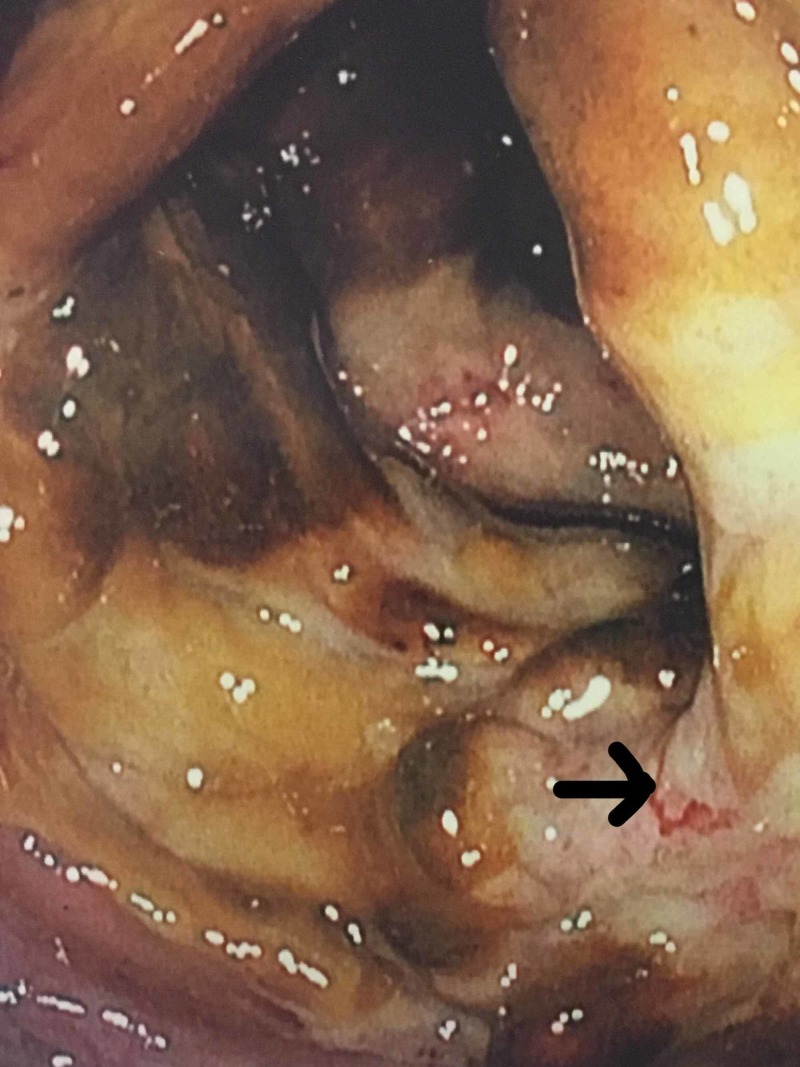
Ulcerated and necrotic cecum with areas of mucosal hemorrhage (arrow)

Computed tomography (CT) abdomen and pelvis, with intravenous (IV) contrast at the time of admission, disclosed a 2.9 cm infra-renal abdominal aortic aneurysm (AAA) with mural thrombus as well as high-grade stenosis of the left common iliac artery (LCIA). The celiac artery (CA), superior mesenteric artery (SMA), and superior mesenteric vein (SMV) were reported to be patent. On Day 2 of admission, a right upper quadrant ultrasound (RUQ US) revealed a small amount of gallbladder sludge but no evidence of cholecystitis or choledocholithiasis. The patient was empirically started on metronidazole and ciprofloxacin for possible infectious and/or ischemic colitis. On Day 2 of admission, she developed rectal bleeding and her WBC count increased to 22.3 x10^3/uL. On Day 3 of admission, a repeat CT abdomen and pelvis with IV contrast was unremarkable. She underwent EGD and colonoscopy on Day 5 of admission. The EGD revealed scattered, atypical appearing ulcers in the fundus, in the body along the lesser curvature, and in the antrum (Figure 1). Slight oozing of blood was seen from several of the ulcers. The esophagus was normal appearing, and the duodenum appeared normal to the second portion. Biopsies were obtained from several ulcerated areas of the fundus and antrum. Histology was notable for marked severe inflammatory exudates suggestive of ischemia. The pathological evaluation did not reveal any evidence of malignancy or Helicobacter pylori infection. Colonoscopy revealed a diffusely ulcerated cecum producing a mass-like appearance and areas of mucosal hemorrhaging were observed (Figure 2).

Biopsies of the cecum revealed inflammatory exudates as well as significant gangrenous necrosis consistent with ischemia. The colon was otherwise normal aside from mild sigmoid diverticulosis. Also obtained on Day 5 of admission was a duplex US of the abdomen, which was remarkable for a peak systolic velocity of 543 cm/s at the origin of the celiac artery, indicating high-grade stenosis. A peak systolic velocity of 805 cm/s at the origin of the SMA was seen, also indicating high-grade stenosis. The origin of the IMA was occluded but evidence of collateral vessel formation was identified distally. The peak systolic velocity at non-occluded portions of the IMA was 52 cm/s. Two days later, the patient underwent a mesenteric angiogram and stenting of the CA and SMA. Dual antiplatelet therapy with aspirin and clopidogrel was initiated and the patient was also started on a statin. On post-procedure Day 1, the patient reported an improvement in her abdominal pain. On post-procedure Day 2, she had a recurrence of her RLQ abdominal pain and developed tachycardia, worsening leukocytosis, and an acute kidney injury. CT abdomen and pelvis without contrast revealed the development of moderate ascites but did not show any evidence of acute bowel injury. On hospital Day 9, the patient underwent exploratory laparotomy. Upon opening the abdomen, 1.5 liters of ascitic fluid was drained. The right colon and cecum were adherent to the anterior pelvic sidewall. Significant swelling of the cecum was seen and a contained perforation was found. The patient underwent a right hemicolectomy with primary anastomosis. The patient tolerated the procedure well and had no complications. The patient was discharged in good condition on hospital Day 19.

## Discussion

Gastric ischemia and cecal ischemia are significant causes of morbidity and mortality, especially in the elderly population [5]. The etiologies of cecal ischemia include obstructive causes as well as non-obstructive ones. Obstructive causes are most commonly due to atherosclerosis, thromboembolism, venous occlusion, as well as mechanical bowel obstruction [3,5]. Non-obstructive causes of cecal ischemia include low perfusion states, which lead to cecal ischemia by a decrease in blood flow and by the triggering of mesenteric vasoconstriction [5-8]. Gastric ischemia likewise arises from diffuse or localized vascular insufficiency [4-5].

Isolated cecal ischemia most commonly presents as either chronic or acute right lower quadrant abdominal pain [9]. In severe isolated gastric ischemia, the patient can develop nausea and vomiting, upper gastrointestinal (GI) bleeding, abdominal pain, and abdominal distention. In cases of chronic gastric ischemia, abdominal discomfort, nausea and vomiting, occult or obvious GI blood loss, weight loss, anemia, and diarrhea can also be the presenting complaints [5,10-11].

Gastric and cecal ischemia can be detected by endoscopic evaluation, cross-sectional imaging, ultrasound examination, and diagnostic angiography [2,5,11-14]. Endoscopy can provide early diagnostic clues, and it also allows for the assessment of the extent of gastric ischemia [5,11,15]. There are typical endoscopic findings in patients with gastric ischemia [11,16]. These findings include scattered gastric mucosal disruptions, obliteration of the vascular pattern in the mucosa, and the presence of ulcers [16-17]. Isolated cecal ischemia can also be difficult to diagnose because it is a relatively rare condition [18]. To arrive at a timely diagnosis, a high clinical suspicion early in the presentation is necessary [2,5]. The use of colonoscopy for suspected ischemic colitis is controversial [5,19]. Its use as a diagnostic modality could exacerbate the condition and cause colonic perforation [5,19]. Nevertheless, if there is a high clinical suspicion and the diagnosis remains in question, colonoscopic evaluation should be considered.

The treatment of gastric ischemia includes fluid resuscitation, low-intermittent nasogastric suction to combat gastric distension, intravenous proton pump inhibitor therapy, and the select usage of broad-spectrum antibiotics if sepsis or gastric pneumatosis is present. Angiographic interventions may be indicated in cases of splanchnic vascular obstruction. Surgical intervention is indicated in cases of gastric perforation, gastric volvulus, or cases of severe gastric ischemia, which fail to improve with medical therapy [1,20]. Currently, there are no established clinical guidelines for the management of gastric ischemia. Despite aggressive management, gastric ischemia overall portends a poor prognosis [1]. In regards to isolated cecal ischemia, if there is suspicion for necrosis of the cecum then exploratory laparotomy should be carried out without delay. The treatment of choice for isolated cecal ischemia with necrosis is cecal resection or right hemicolectomy [5,7]. Patients with isolated cecal necrosis have a good prognosis with early diagnosis and surgical management [5].

## Conclusions

Gastric ischemia is rare due to the rich vascular supply of the stomach. However, gastric ischemia is likely underdiagnosed. The endoscopic findings of scattered or localized gastric mucosal disruptions, obliteration of the vascular pattern in the mucosa, and the presence of atypical-appearing ulcers should raise the suspicion for gastric ischemia and prompt treatment should be initiated.

Clinicians should consider isolated cecal ischemia with necrosis in patients presenting with significant RLQ abdominal pain, especially those with underlying vascular disease. While isolated cecal ischemia with necrosis has high morbidity and mortality, with early diagnosis and prompt treatment, it can be successfully managed
